# Potential Effect of Etoricoxib in Reducing Inflammation in Methotrexate-Induced Pulmonary Injury in Rats: Role of Oxidative Stress and the TLR4/p38-MAPK/NF-κB Signaling Pathway

**DOI:** 10.1007/s10753-024-02198-w

**Published:** 2024-11-27

**Authors:** Ali M. Ali Abdelall, Ali Khames, Amany Abdlrehim Bekhit, Moustafa Fathy

**Affiliations:** 1 Department of Biochemistry, Faculty of Pharmacy, Sohag University, Sohag, 82511 Egypt; 2 Department of Pharmacology and Toxicology, Faculty of Pharmacy, Sohag University, Sohag, 82511 Egypt; 3 Department of Biochemistry, Faculty of Pharmacy, Minia University, Minia, 61519 Egypt; 4 Department of Regenerative Medicine, Graduate School of Medicine and Pharmaceutical Sciences, University of Toyama, Toyama, 930-0194 Japan

**Keywords:** Pulmonary toxicity, MTX, ETO, Inflammation, Oxidative stress, NF-κβ, TLR-4

## Abstract

Numerous chemotherapeutic medications can have hazardous effects on the lungs, which can result in severe lung diseases. Methotrexate (MTX) is prescribed for cancer and inflammation-related disorders; nevertheless, it is exceptionally highly toxic and has multiple kinds of adverse reactions, including pulmonary injury. Our work was designed to demonstrate the ability of etoricoxib (ETO) to mitigate MTX-induced lung injury in experimental animals. Adult male Wistar rats were separated into four groups. The first group consisted of healthy controls that received carboxymethyl cellulose (1 ml/day, p.o.), the second group received a single dose of MTX (20 mg/kg/day, *i.p.*), the third group received ETO (10 mg/kg/day, p.o.) for three weeks, and the fourth group first received a single MTX (20 mg/kg, i.p.) and then was treated with ETO for three weeks. Concomitant treatment with ETO and MTX improved the histological structure of the lung tissue. It significantly altered the levels of oxidant/antioxidant markers, such as malondialdehyde (MDA), heme oxygenase-1 (HO-1), reduced glutathione (GSH), and nuclear factor erythroid 2-related factor 2 (Nrf-2), in favor of antioxidants. Moreover, ETO can normalize the proinflammatory cascade, which includes tumor necrosis factor-alpha (TNF-α) and interleukin-1 beta (IL-1β). At the molecular level, ETO downregulated the protein expression of toll-like receptor 4 (TLR4), nuclear factor kappa-B (NF-κB), and p38 mitogen-activated protein kinase (p38 MAPK) in inflamed rat lungs. In conclusion, our findings indicate that oral administration of ETO ameliorates MTX-induced lung injury by inhibiting oxidative stress and suppressing the TLR4/NF-κB and TLR4/p38-MAPK inflammatory signaling pathways.

**Supplementary Information:**

The online version contains supplementary material available at 10.1007/s10753-024-02198-w.

## 1-Introduction

Chemotherapeutic drugs are commonly utilized to treat hematologic and solid tumors [1, 2]. Methotrexate (MTX) is a folate antagonist that is widely prescribed for multiple types of tumors as well as nonneoplastic conditions, including psoriasis and rheumatoid arthritis [3, 4]. Nevertheless, most patients treated with MTX are forced to discontinue treatment because of serious side effects, such as liver, lung, kidney, and brain toxicities [5, 6]. The mechanisms underlying MTX-induced liver and lung damage have been the subject of numerous studies. The depletion of antioxidant defenses because of the generation of reactive oxygen species (ROS) is one of these mechanisms, and it can lead to parenchymal lung injury, interstitial lung disease, alveolar fibrosis, and hepatotoxicity [7–9]. TLR4, a member of the TLR family, activates the nuclear factor-kappa B (NF-κB) pathway, increasing the production of proinflammatory cytokines such as tumor necrosis factor-α (TNF-*α*) and interleukin-6 (IL-6) [10]. NF-κB is a transcription factor of notable value that causes inflammation and lung damage. p38 mitogen-activated protein kinase (p38-MAPK) is also an essential signaling pathway in MTX-induced lung injury that facilitates the release of cytokines and promotes inflammation [11]. While NF-κB and p38-MAPK play active roles in lung damage, blocking these pathways could provide a new approach to manage lung injury [12, 13].

Etoricoxib (ETO), a novel selective COX-2 inhibitor, is an influential member of the COX-2 NSAID selective family with minimal side effects in the gastrointestinal tract (GIT) [14, 15]. In addition to its ability to improve safety and tolerance, it also has anti-inflammatory and analgesic properties in the treatment of osteoarthritis, acute gouty arthritis, ankylosing spondylitis, primary dysmenorrhea, rheumatoid arthritis, low back pain, and acute postoperative pain [16–18]. According to a recent report, ETO considerably reduces the risk of oxidative stress associated with cerebral ischemia [19]. Moreover, it can protect testicles from oxidative damage caused by torsion detorsion [20]. Previous studies have shown that selective inhibition of COX-2 can halt ischemic neuronal injury in rats [21]. Since MTX treatment has been known to cause a wide range of side effects in some cases, our review of the published data revealed no information on ETO activity against pulmonary complications established by MTX in rats. Thus, this work was conducted to assess the effect of the selective COX-2 inhibitor ETO on MTX-induced lung damage and elucidate the relevant pathway in rats.

## 2-Materials and methods

### Animal models

We employed forty-eight adult male rats of the Sprague–Dawley strain weight (approximately 180 ± 5.0 g) provided by Sohag University, Faculty of Medicine, Animal House Facility. The treatment was started after the acclimatization of the rats for two weeks at 25 °C ± 0.5 °C with a 12-h dark/light cycle. The animals were maintained in clean polycarbonate cages for the trial and had unlimited access to a standard chow pellet diet and tap water ad libitum. The Research Ethical Committee of the Faculty of Pharmacy, Sohag University (Sohag, Egypt), recommended that experimental methods be carried out in compliance with the ethical standards for studies involving experimental rats and to follow the rules of the Care and Use of Experimental Rats (ILAR, 1996).

## Drugs and chemicals

MTX was obtained from Minaspharm Pharmaceuticals (Cairo, Egypt), and etoricoxib (Egyphar Co., Obour City, Egypt), reduced glutathione (GSH) (Cat. No: GR 2511) and malondialdehyde (MDA) (Cat. No: MD2529) were purchased from Biodiagnostic, Cairo, Egypt. ELISA kits for heme oxygenase-1 (HO-1) (catalog no: MBS 764989) and nuclear factor erythroid 2 related factor 2 (NFE2R2) (catalog no: MBS 752046) were obtained from My BioSource (San Diego, CA, USA). Rat tumor necrosis factor-alpha (TNF-α) ELISA Kit (Cusabio Life Sciences, Wuhan, China; Catalog No: CSB-E11987r) was used. Rat IL-1β ELISA Kit (My BioSource, San Diego, USA; catalog No: MBS825017). TGX Stain-Free™ FastCast™ Acrylamide Kit (SDS‒PAGE) (Bio-Rad Laboratories Inc., USA; Cat. number: 161–0181).

## Experimental Design

Four groups of forty-eight rats were assigned at random as follows:

1. Control group (n = 12): Rats were given CMC (1 ml/day, p.o.) for three weeks.
2. In the MTX group (n = 12), the rats were given a single intraperitoneal (*i.p*.) dose of MTX (20 mg/kg) [22].
3. ETO group (n = 12): Rats were given ETO (10 mg/kg/day, p.o.) for three weeks [23].
4. In the MTX + ETO group (n = 12), the rats were given a single dose of MTX (20 mg/kg, *i.p.*) on the first day of the trial and then continued with ETO treatment (10 mg/kg/day, p.o.) for three weeks.

## Sample collection and tissue preparation

Twenty-four hours after the last treatment, the rats were anesthetized with (1 g/kg, *i.p.*) urethane. Blood samples were collected by decapitation. The lungs of each animal were immediately excised, washed with cold 0.9% NaCl, dried on filter paper, weighed, and frozen at −80 °C [24]. Lung sections from each group were preserved in 10% formalin for histological analysis [25]. A portion of the lungs was homogenized in cold phosphate-buffered saline (PBS, pH 7.4). The protease inhibitor cocktail (Biospes, China) employs a motor-driven homogenizer (LabGEN 7, Cole-Parmer, USA) to create a 20% w/v homogenate; then, the product was centrifuged at 4000 rpm for 15 min at 4 °C, and the supernatant was stored at −20 °C for further biochemical assessment.

## Biochemical analysis

### Measurement of pulmonary oxidative stress biomarkers

The oxidative stress markers GSH and MDA were measured in lung homogenates via commercially available kits and a spectrophotometer (Win spec, U.S.A.) following the manufacturer's instructions (Biodiagnostic, Egypt). HO-1 and Nrf-2 concentrations were measured in lung homogenates via rat-specific enzyme-linked immunosorbent assay (ELISA) kits (Elisa Plate Reader statfax, USA) according to the manufacturer's instructions (My BioSource, USA).

### Measurement of Pulmonary Proinflammatory Cytokines

The levels of the proinflammatory cytokines TNF-α and IL-1β were determined in a lung homogenate via rat ELISA kits (Elisa Plate Reader stat fax, USA) according to the manufacturer's instructions (My BioSource, USA).

### Measurement of Pulmonary Toll-Like Receptor 4 (TLR-4), p38 Mitogen-Activated Protein Kinases (P38-MAPK), Nuclear Factor Kappa B (NF-κB), and Western Blot Analysis

A TGX Stain-FreeTM Fast Cast™ Acrylamide Kit (SDS‒PAGE) was used to analyze the protein expression levels of TLR-4, NF-κB, and p38-MAPK via Western blotting. In brief, each sample's protein concentration was determined via the Bradford assay according to the manufacturer's instructions [26]. Each sample's protein concentration was then quantified in a volume equal to 20 μg, with an equal volume of 2 × Laemmli sample buffer consisting of 4% SDS, 10% 2-mercaptoethanol, 20% glycerol, 0.004% bromophenol blue, and 0.125 M Tris HCl, which was isolated via sodium dodecyl sulfate‒polyacrylamide gel electrophoresis. According to the manufacturer's instructions, polyacrylamide gels were generated via the TGX Stain-Free™ FastCast™ Acrylamide Kit (Bio-Rad Laboratories, Inc.). After the proteins were separated via SDS‒PAGE, a Bio-Rad Trans-Blot system (Pierce, Rockford, IL) was used to transfer the protein bands to a PVDF membrane (Millipore, Merck, United States). The blot was washed with PBS three times for 5 min, and 5% (w/v) skim milk powder in PBS was used to block the membranes for 1 h at room temperature. At the end of the blocking step, the membranes were incubated overnight at 4 °C with unique antibodies against TLR4, NF-κB, p38-MAPK (1:500) and (1:5000) diluted β-actin and then incubated for 2 h at room temperature with an HRP-conjugated secondary antibody (goat anti-rabbit IgG-HRP mAb; Novus Biologicals) (1:1000). By using Image Lab TM software version 5.1 (Bio-Rad Laboratories, Hercules, California) with the imaging system ChemiDocTM, the band intensity was assessed. The quantification was performed via densitometric analysis via ImageJ 7.0 analysis software (National Institutes of Health, Bethesda, United States) against the loading control β-actin bands [27].

## Histopathological examination

For 24 h, lung samples from all groups were maintained in 10% normal saline. A series of alcohol dilutions (methyl. ethyl and 100% ethyl) were utilized to dehydrate the samples after they were washed with tap water. The samples were purified in xylene before being incubated for 24 h at 56 °C in a hot air oven with paraffin. The tissue blocks of paraffin and beeswax were divided into 4-µm-thick pieces via a rotary LEITZ microtome. The acquired pieces of tissue were placed on glass slides, deparaffinized, and stained with hematoxylin and eosin. Photomicrographs were taken via an Olympus (U.TV0.5XC-3) light microscope and a digital camera [28].

## Statistical analysis

We employed one-way analysis of variance (ANOVA) and Tukey’s multiple comparisons post hoc test to determine statistically significant differences between the groups. GraphPad Prism 8 software (GraphPad Software, Inc.) was used to create the graphics and conduct the statistical investigation. To express the data, the mean ± SD was used. Differences were considered significant when the *p* value was less than or equal to 0.05.

## Results

### Effects of etoricoxib on pulmonary oxidative stress markers in rats with MTX-induced lung toxicity

**Fig. 1:**
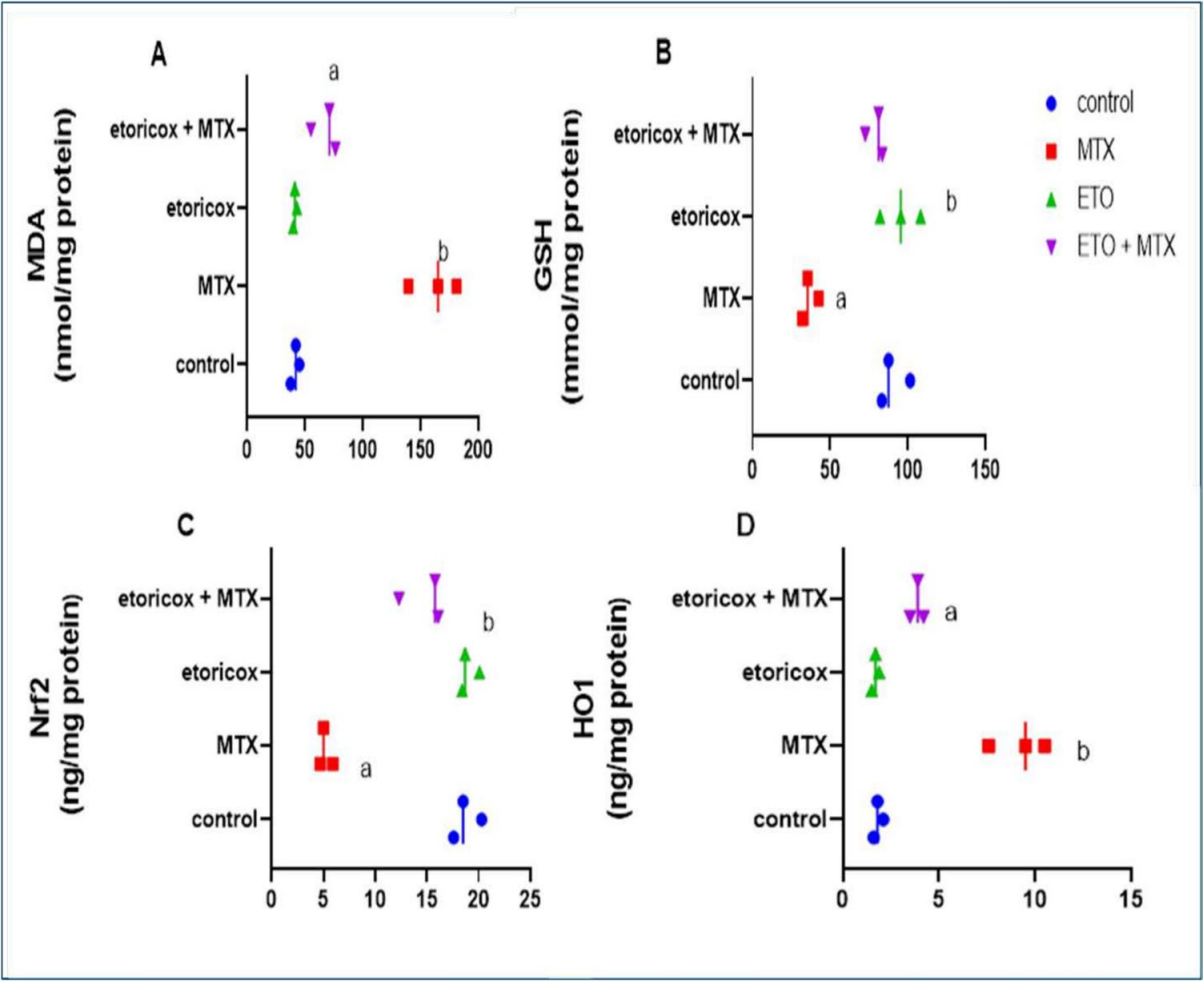
Effects of ETO on the pulmonary oxidative stress markers A) MDA content, B) GSH level, C) NF-2 activity, and D) HO-1 activity in MTX-treated rats. The data are presented as the means ± S.Ds. (n = 12 mice). a, b Significantly different from the normal control and MTX groups, respectively, at *P* < *0.05*. MTX: methotrexate; ETO: etoricoxib; MDA: malondialdehyde; GSH: reduced glutathione; NF-2: nuclear factor erythroid 2-related factor 2; HO-1: heme oxygenase-1

The present data revealed that the administration of MTX strongly increased oxidative stress, as indicated by significant (*P* < *0.05*) increases in the HO-1 activity and MDA content in the lung tissue of the MTX-treated group compared with those in the control group. In addition, there was a substantial (*P* < 0.05) decrease in the GSH content and Nrf-2 activity in the lung tissue of the MTX group compared with those of the control group. Interestingly, the group given ETO orally following MTX injection presented a significant (*P* < *0.05*) reduction in the lung MDA content and HO-1 activity and entirely recovered (*P* < *0.05*) in the lung content of GSH and the activity of Nrf-2 relative to those of the MTX group (Fig. 1. A-D).

## Effect of etoricoxib on pulmonary proinflammatory cytokines in rats with MTX-induced lung toxicity

**Fig. 2:**
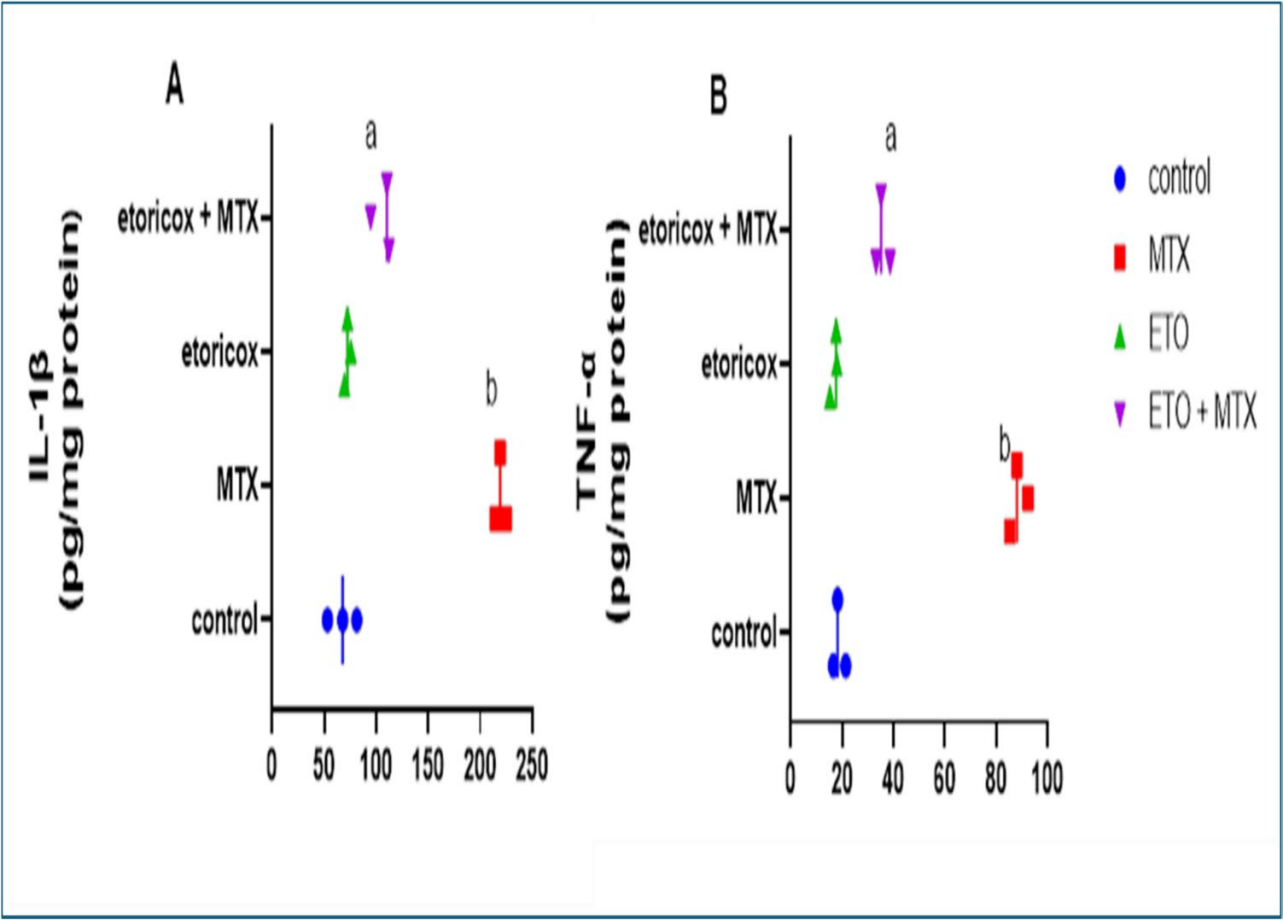
Effects of ETO on the levels of the pulmonary proinflammatory markers A) IL-1B and B) TNF- in MTX-treated rats. The data are presented as the means ± S.Ds. (n = 12 mice). a, b Significantly different from the normal control and MTX groups, respectively, at *P* < *0.05*. MTX: methotrexate; ETO: etoricoxib; TNF-α: tumor necrosis factor-alpha; IL-1β: interleukin-1 beta

Compared with those of control rats, the lungs of MTX-treated rats presented significant (*P* < *0.05*) increases in the levels of the proinflammatory cytokines TNF-α and IL-1β. In contrast, rats previously treated with MTX presented significantly (*P* < *0.05*) lower TNF-α and IL-1β levels after receiving ETO therapy than did those in the MTX group (Fig. 2. A-C).

## Effect of etoricoxib on pulmonary TLR4, P38-MAPK, and NF-κB protein levels in rats with MTX-induced lung toxicity

**Fig. 3:**
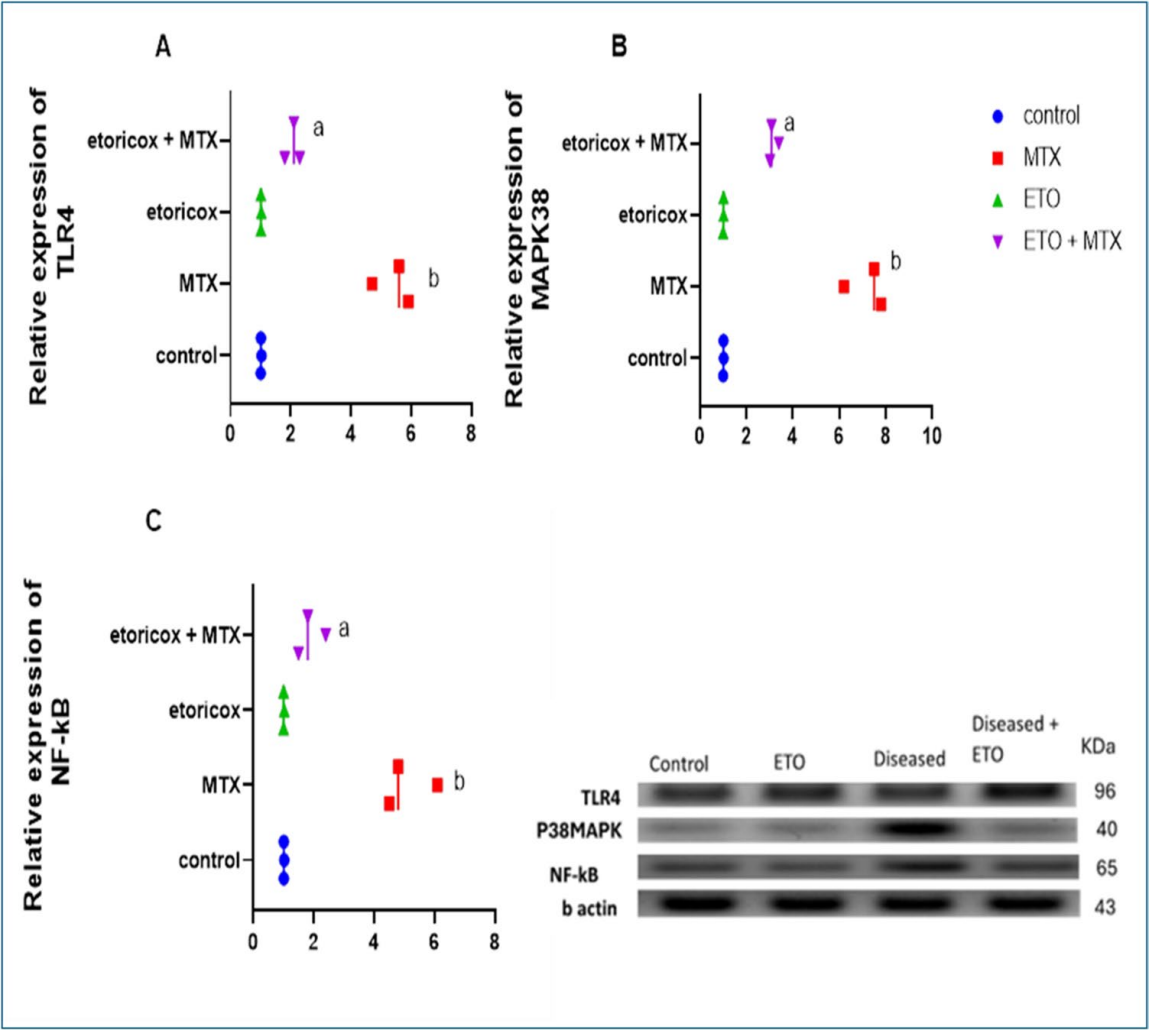
Effects of ETO on pulmonary protein expression levels of A) TLR4, B) p38MAPKN, and C) NF-kB in MTX-treated rats. The data are presented as the means ± S.Ds. (n = 12 mice). a, b Significantly different from the normal control and MTX groups, respectively, at *P* < *0.05*. MTX: methotrexate; ETO: etoricoxib; TLR4: toll-like receptor 4; p38 MAPK: p38 mitogen-activated protein kinase; NF-κB: nuclear factor kappa-B

Compared with control rats, MTX-treated rats presented significantly *(P* < *0.05*) greater TLR4, p38-MAPK, and NF-κB protein expression levels in lung tissues. In contrast to the group treated with MTX, the group that received ETO therapy following MTX administration presented significant (*P* < *0.05*) reductions in the protein expression levels of TLR4, p38 MAPK, and NF-κB in the lung tissue of experimental animals (Fig. 3. A-B).

## Effect of etoricoxib on the histopathological structure of pulmonary tissues in MTX-treated rats

**Fig. 4:**
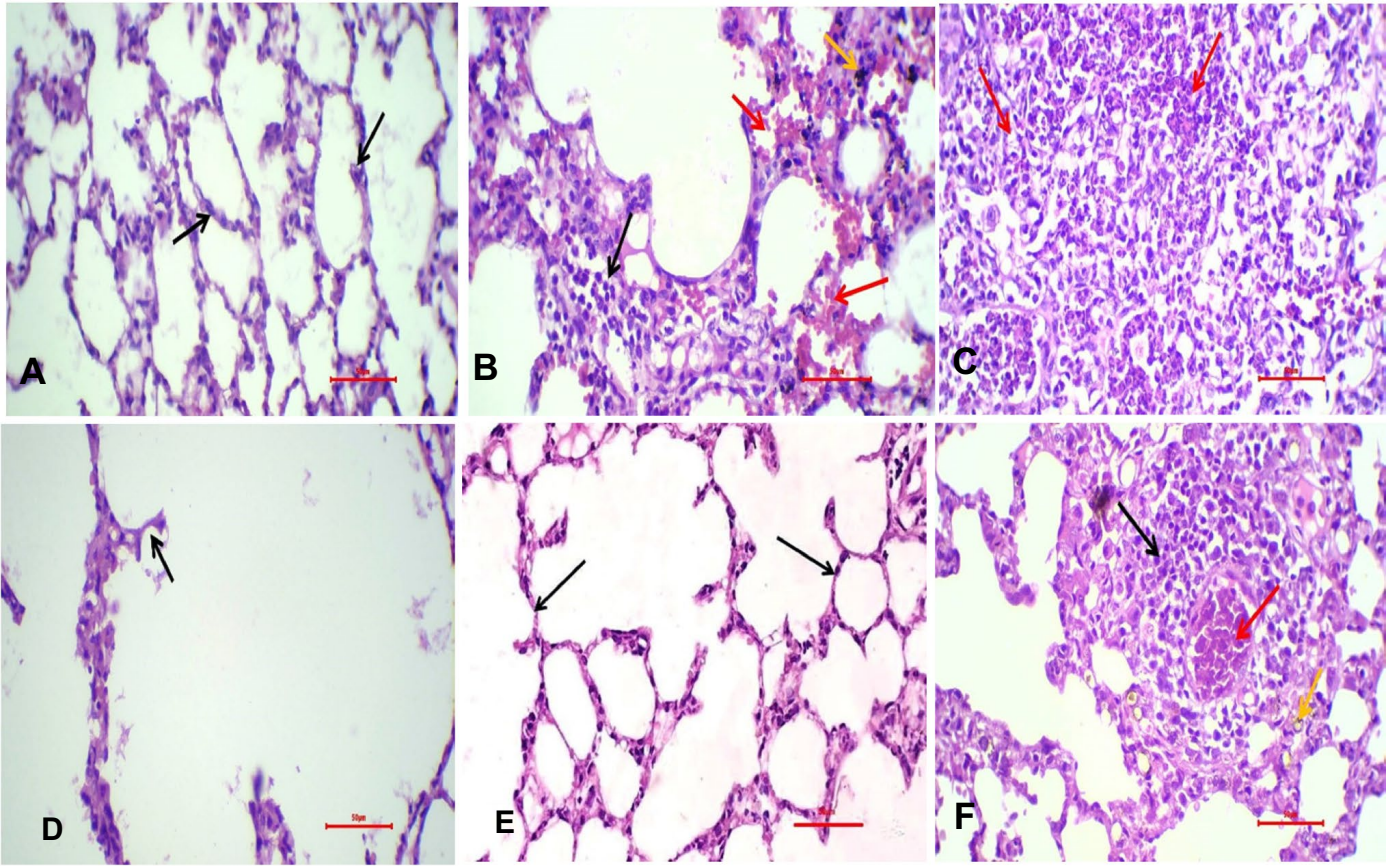
Effects of ETO on pulmonary histopathological alterations in MTX-treated rats (n = 12 rats) examined via H&E staining. **(A)** A photomicrograph (× 400) of lung sections from the control group showing preserved alveolar structures. The alveoli are rather uniform in size and shape. The alveolar walls have average thickness with no thickening, edema, or inflammation (black arrows). (**B-D)** A photomicrograph (× 400) of the lung tissue of MTX-treated rats showing widespread interstitial and intra-alveolar hemorrhage (yellow arrow). Numerous congested capillaries of the alveolar wall were observed. Focal dense infiltration by mixed inflammatory cells, including neutrophils and lymphocytes (red arrows), was detected. Numerous hemosiderin-laden macrophages and hemosiderin particles were observed. Focal emphysema with disruption of the alveolar wall was detected (black arrow). **(E)** A photomicrograph (× 400) of lung sections from the ETO-treated group shows a lung structure like that of the negative control group (black arrows). **(F)** A photomicrograph (× 400) of lung sections from MTX-treated rats subjected to ETO showing mild residual inflammation of the lung tissue with relative thickening of the alveolar wall (black arrow). Mild residual congestion (red arrow). Mild residual hemorrhage and a few hemosiderin particles were also detected (yellow arrow)

Sections of lungs from the control and ETO groups revealed no detectable histological modifications in the structure of the bronchioles or air alveoli surrounding them (Fig. 4A). Meanwhile, the MTX-treated group displayed significant peribronchiolar inflammatory cell aggregation, infiltration linked to peribronchiolar and perialveolar blood vessel congestion, and alveolar emphysema (Fig. 4B‒D). Compared with the control group, the group in which only ETO was administered did not exhibit any structural alterations in the lung tissue sections. When the animals received ETO after MTX injection, the lung tissue sections presented mildly thickened walls and slight infiltration of inflammatory cells in the peribronchiolar region (Fig. 4, F) (Table 1).

**Table 1:** The findings of degree of fibrosis (Ashcroft scale) and histopathological scoring

		*Degree of fibrosis (Ashcroft scale)	**Histopathological scoring				
			Bronchitis/ bronchiolitis	Edema	Hemorrhage	Thickening of alveolar wall	Degeneration of alveolar and bronchial cells
1	Group 1 (carboxymethyl cellulose—negative control)	0	0	0	0	0	0
2	Group 2 (methotrexate treated rats—positive control)	4	3	4	3	4	3
3	Group 3 (etoricoxib administered rats):	0	0	0	0	0	0
4	Group 4 (experimentally inducted rats and administered etoricoxib)	1	1	1	0	1	1

*Degree of fibrosis (Ashcroft scale) according to Hübner, et. Al. (2008)

**Histopathological scoring. * Tissue injury of lung tissue was scored in degrees as follows: 0 = no change; 1 = < 25% tissue damage; 2 = 26–50% tissue damage; 3 = 51–75% tissue damage; 4 = 76–100% tissue damage

**Hubner RH, Gitter W, El Mokhtari NE, et al.** Standardized quantification of pulmonary fibrosis in histological samples. Biotechniques. 2008;44:507–511, 514–517

**Gibson-Corley KN, Olivier AK, Meyerholz DK (2013)** Principles for valid histopathologic scoring in research. Vet Pathol 50:1007–1015

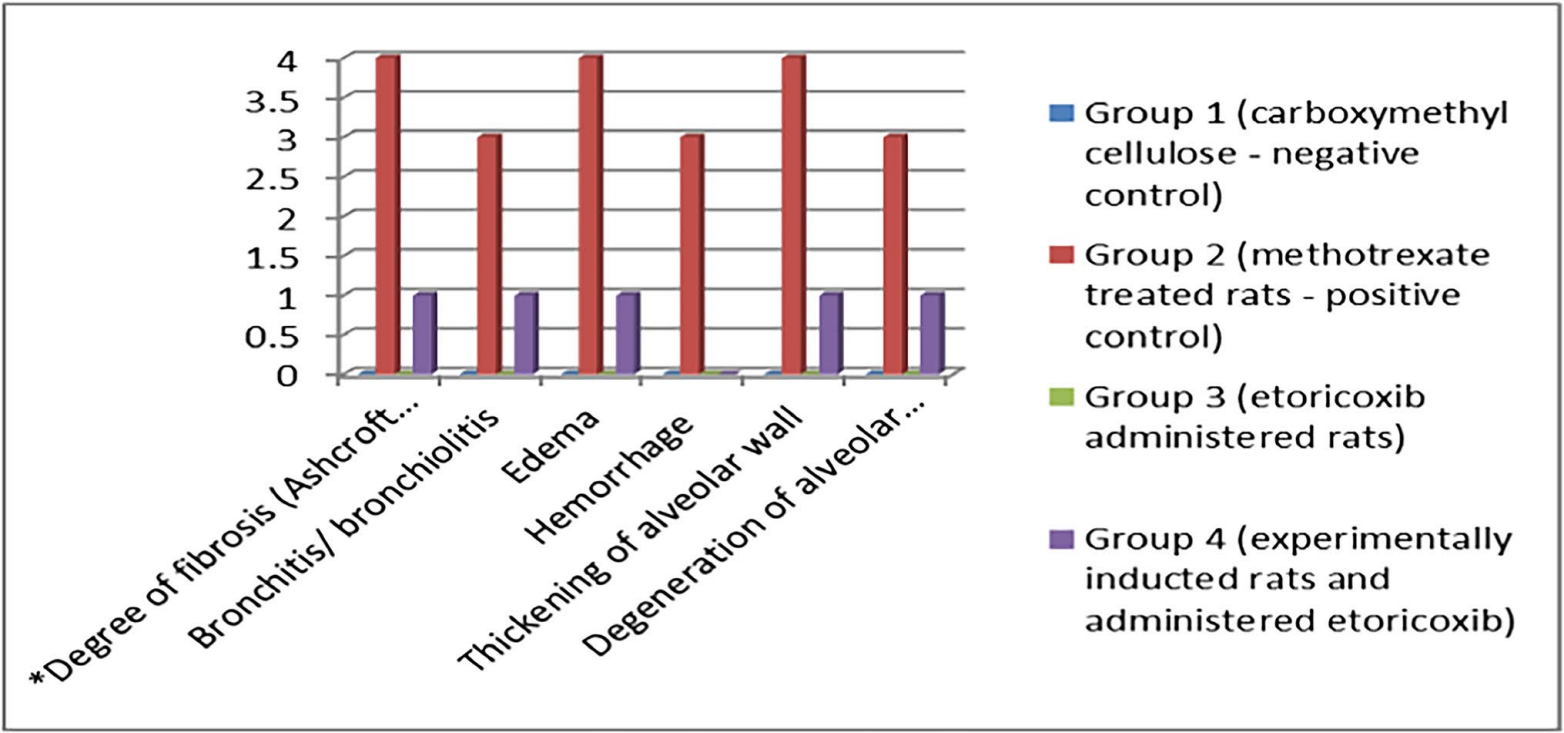

## Discussion

COX-2 is an influential rate-limiting enzyme in the biosynthesis of inflammatory mediators such as PGs; thus, hindering COX activity is the initial therapeutic action of NSAIDs to treat acute and chronic inflammatory diseases [29].

This study revealed that ETO, selective COX-2 inhibitor. alleviates MTX-induced lung injury in rats, as evidenced by histological and biochemical assessments, and this finding is related to the prevention of oxidative stress and inflammation. MTX is recommended for acute leukemia or severe psoriasis; however, lung toxicity is one of its limitations [30]. Its antiproliferative properties are obtained by inhibiting DNA biosynthesis and important elements of the immune system, especially T cells. Additionally, it may exacerbate inflammatory reactions by activating NF-κB and activator protein-1 through the MAPK pathway [31].

The present work suggested that the oxidative marker MDA content and HO-1 activity were significantly elevated, whereas the total GSH content and Nrf-2 activity in the lungs were substantially decreased after a single MTX dose. These outcomes are reliable in previous studies [32–35].

ROS are critical molecules in multifunctional signalling cascades. According to reports, elevated ROS causes HO-1 induction, Nrf2 nuclear translocation, and the MAPK signalling pathways [36, 37]. Similarly, Nrf-2, a redox-sensitive basic-leucine zipper transcription factor, relocates to the nucleus upon oxidative stress leading to trigger the synthesis of cytoprotective and defensive molecules such as GSH [38, 39]. Heme oxygenase (HO)−1 activation represents a crucial cytoprotective strategy to maintain cellular homeostasis during inflammation [40]. MTX clearly causes pulmonary toxicity since it directly affects ROS generation, which in turn increases the level of MDA, a polyunsaturated fatty acid product that indicates lipid peroxidation, along with HO-1, an essential enzyme involved in antioxidant processes [41, 42]. Therefore, the oxidant/antioxidant imbalance due to the overproduction of ROS during MTX treatment is one of the primary causes of MTX-induced lung injury [43–45]. Our investigation found that MDA and HO-l levels decreased significantly after ETO treatment, whereas GSH and Nrf-2 levels increased. Furthermore, a previous study reported that the 2nd generation selective COX-2 inhibitor ETO protected rats from adverse effects of HF diet-induced oxidative stress with a concurrent increase in the activity of antioxidant enzymes [18]. These effects are believed to be attributed mainly to the inhibitory action of ETO on COX-2 and ROS production [46, 47].

The levels of proinflammatory cytokines, such as TNF-α and IL-1β, are key indicators of inflammation in MTX-treated rats. Monocytes and macrophages produce the main inflammatory cytokines IL-1β and TNF-α in response to intrinsic and extrinsic stimuli. Later, leucocytes are provoked to migrate from blood vessels and gather at sites of damage or infection [48]. Proinflammatory and anti-inflammatory cytokines are in balance in healthy lung tissues. However, in the context of MTX-induced lung damage, the balance shifted in favor of proinflammatory cytokines, as proven by markedly elevated levels of TNF-α and IL-1β in lung tissues [48–50]. These outcomes are in line with our experimental findings. In healthy tissues, COX-2 synthesis is low, but in inflammatory tissues, COX-2 activity is noticeably increased [51, 52]. Our results are consistent with earlier findings that revealed that ETO inhibited an increase in the levels of IL-1β and TNF-α, which could be related to the decrease in the inflammatory activity of COX-2 [15, 53].

Notably, several signaling cascades, such as Toll-like receptor 4 (TLR4), nuclear factor-kappa B (NF-κB), and activator of transcription (JAK/STAT), and the mitogen-activated protein kinase (MAPK) pathway, are involved in the modulation of inflammatory responses in the context of MTX-induced tissue damage [54, 55]. To gain a deeper understanding of the favorable effect of ETO on MTX-induced lung injury, we focused on the TLR4/p38-MAPK/NF-κB signaling pathway, which plays a crucial role in coordinating various cellular activities [56].

Our results indicated that MTX drastically increased the protein levels of TLR-4, NF-κB and p38-MAPK in the lungs of experimental animals compared with those in the lungs of control rats. Surprisingly, we found that ETO could abrogate the marked increase in those proteins. Numerous inflammatory mediators as well as ROS stimulate COX-2, but certain redox-sensitive transcription factors, such as NF-κB, MAPK kinase, and p38 kinase, tightly control its expression, at least in part [57]. Also, MAPK pathways have been proven to be involved in HO-1 expression [58]. ETO has an anti-inflammatory effect, as evidenced by the reduction in COX-2 protein expression in rats treated with DMH [59]. Thus, we support prior work suggesting that the noticeable decrease in these proteins could be attributed to the COX-2 inhibitor being received [60]. In parallel, the results of the lung tissue histopathology analysis provided additional support for our findings. In the context of MTX-induced lung structural damage, ETO significantly reduced interstitial and intra-alveolar hemorrhage, congested capillaries of the alveolar wall, and inflammatory cells, including neutrophils and lymphocytes.

## Conclusion

Overall, ETO, a COX-2 selective inhibitor, demonstrates superior anti-inflammatory and antioxidant effects in MTX-induced lung injury in rats via reactive oxygen species (ROS)-related Nrf2/HO-1 and TLR-4/p38/MAPK NF-kB/IL-1β/TNF-α signalling. These findings provide a novel mechanistic explanation for ETO's anti-inflammatory properties.

## Supplementary Information

Below is the link to the electronic supplementary material.

Supplementary file1 (PDF 129 KB)

## Data Availability

No datasets were generated or analysed during the current study.

## References

[1] Limper, A.H. 2004. Chemotherapy-induced lung disease. *Clinics in Chest Medicine* 25 (1): 53–64. 15062597 10.1016/S0272-5231(03)00123-0

[2] Alfwuaires, M.A. 2022. Galangin mitigates oxidative stress, inflammation, and apoptosis in a rat model of methotrexate hepatotoxicity. *Environmental Science and Pollution Research International* 29 (14): 20279–20288. 34729716 10.1007/s11356-021-16804-z

[3] Ohbayashi, M., et al. 2014. Involvement of epithelial-mesenchymal transition in methotrexate-induced pulmonary fibrosis. *Journal of Toxicological Sciences* 39 (2): 319–330. 24646714 10.2131/jts.39.319

[4] Elsawy, H., et al. 2022. Beneficial role of naringin against methotrexate-induced injury to rat testes: Biochemical and ultrastructural analyses. *Redox Report* 27 (1): 158–166. 35861275 10.1080/13510002.2022.2101832 PMC9310850

[5] Abdalhameid, E., et al. 2024. Cinnamic acid mitigates methotrexate-induced lung fibrosis in rats: Comparative study with pirfenidone. *Naunyn-Schmiedeberg’s Archives of Pharmacology* 397 (2): 1071–1079. 37581637 10.1007/s00210-023-02652-w PMC10791841

[6] Chen, J., et al. 2021. Dexmedetomidine reverses MTX-induced neurotoxicity and inflammation in hippocampal HT22 cell lines via NCOA4-mediated ferritinophagy. *Aging (Albany NY)* 13 (4): 6182–6193. 33632938 10.18632/aging.202626 PMC7950253

[7] Serrano-Mollar, A., et al. 2003. In vivo antioxidant treatment protects against bleomycin-induced lung damage in rats. *British Journal of Pharmacology* 138 (6): 1037–1048. 12684259 10.1038/sj.bjp.0705138 PMC1573750

[8] Isik, M., et al. 2017. Oxidative stress and mRNA expression of acetylcholinesterase in the leukocytes of ischemic patients. *Biomedicine & Pharmacotherapy* 87: 561–567. 28081467 10.1016/j.biopha.2017.01.003

[9] Al Maruf, A., et al. 2018. Methotrexate induced mitochondrial injury and cytochrome c release in rat liver hepatocytes. *Drug and Chemical Toxicology* 41 (1): 51–61. 28298149 10.1080/01480545.2017.1289221

[10] Pan, W.Z., et al. 2018. The roles of NF-kB in the development of lung injury after one-lung ventilation. *European Review for Medical and Pharmacological Sciences* 22 (21): 7414–7422. 30468489 10.26355/eurrev_201811_16281

[11] Koppelmann, T., et al. 2021. The Mechanisms of the Anti-Inflammatory and Anti-Apoptotic Effects of Omega-3 Polyunsaturated Fatty Acids during Methotrexate-Induced Intestinal Damage in Cell Line and in a Rat Model. *Nutrients* 13 (3): 888. 33801889 10.3390/nu13030888 PMC8000946

[12] Yu, H.L., et al. 2015. Pinellia ternata lectin exerts a pro-inflammatory effect on macrophages by inducing the release of pro-inflammatory cytokines, the activation of the nuclear factor-kappaB signaling pathway and the overproduction of reactive oxygen species. *International Journal of Molecular Medicine* 36 (4): 1127–1135. 26310942 10.3892/ijmm.2015.2315

[13] Sayed, A.M., et al. 2022. Targeting inflammation and redox aberrations by perindopril attenuates methotrexate-induced intestinal injury in rats: Role of TLR4/NF-kappaB and c-Fos/c-Jun pro-inflammatory pathways and PPAR-gamma/SIRT1 cytoprotective signals. *Chemico-Biological Interactions* 351: 109732. 34737150 10.1016/j.cbi.2021.109732

[14] Takemoto, J.K., et al. 2008. Clinical pharmacokinetic and pharmacodynamic profile of etoricoxib. *Clinical Pharmacokinetics* 47 (11): 703–720. 18840026 10.2165/00003088-200847110-00002

[15] Kunak, C.S., et al. 2015. The Effect of Etoricoxib on Hepatic Ischemia-Reperfusion Injury in Rats. *Oxidative Medicine and Cellular Longevity* 2015: 598162. 26236425 10.1155/2015/598162 PMC4506808

[16] Brooks, P., and P. Kubler. 2006. Etoricoxib for arthritis and pain management. *Therapeutics and Clinical Risk Management* 2 (1): 45–57. 18360581 PMC1661646

[17] Rodrigues, A.D., et al. 2003. Absorption, metabolism, and excretion of etoricoxib, a potent and selective cyclooxygenase-2 inhibitor, in healthy male volunteers. *Drug Metabolism and Disposition* 31 (2): 224–232. 12527704 10.1124/dmd.31.2.224

[18] Kabir, F., et al. 2021. Etoricoxib treatment prevented body weight gain and ameliorated oxidative stress in the liver of high-fat diet-fed rats. *Naunyn-Schmiedeberg’s Archives of Pharmacology* 394 (1): 33–47. 32780227 10.1007/s00210-020-01960-9

[19] Maheshwari, A., et al. 2011. Protective effect of Etoricoxib against middle cerebral artery occlusion induced transient focal cerebral ischemia in rats. *European Journal of Pharmacology* 667 (1–3): 230–237. 21635885 10.1016/j.ejphar.2011.05.030

[20] Yapanoglu, T., et al. 2017. Effect of etoricoxib on experimental oxidative testicular ischemia-reperfusion damage in rats induced with torsion-detorsion. *Korean J Physiol Pharmacol* 21 (5): 457–464. 28883750 10.4196/kjpp.2017.21.5.457 PMC5587596

[21] Nogawa, S., et al. 1997. Cyclo-oxygenase-2 gene expression in neurons contributes to ischemic brain damage. *Journal of Neuroscience* 17 (8): 2746–2755. 9092596 10.1523/JNEUROSCI.17-08-02746.1997 PMC6573095

[22] Arpag, H., et al. 2018. Protective effects of alpha-lipoic acid on methotrexate-induced oxidative lung injury in rats. *Journal of investigative surgery* 31 (2): 107–113. 28340320 10.1080/08941939.2017.1296513

[23] Abd El-Kader, M., and R.I. Taha. 2020. Comparative nephroprotective effects of curcumin and etoricoxib against cisplatin-induced acute kidney injury in rats. *Acta Histochemica* 122 (4): 151534. 32151374 10.1016/j.acthis.2020.151534

[24] Botros, S.R., et al. 2024. Comparative effects of incretin-based therapy on doxorubicin-induced nephrotoxicity in rats: The role of SIRT1/Nrf2/NF-kappaB/TNF-alpha signaling pathways. *Frontiers in Pharmacology* 15: 1353029. 38440177 10.3389/fphar.2024.1353029 PMC10910313

[25] El-Sheikh, A.A., M.A. Morsy, and A.H. Hamouda. 2016. Protective Mechanisms of Thymoquinone on Methotrexate-induced Intestinal Toxicity in Rats. *Pharmacognosy Magazine* 12 (Suppl 1): S76-81. 27041864 10.4103/0973-1296.176106 PMC4792005

[26] Bradford, M.M. 1976. A rapid and sensitive method for the quantitation of microgram quantities of protein utilizing the principle of protein-dye binding. *Analytical Biochemistry* 72: 248–254. 942051 10.1016/0003-2697(76)90527-3

[27] Shehata, A.H.F., et al. 2020. The impact of single and combined PPAR-alpha and PPAR-gamma activation on the neurological outcomes following cerebral ischemia reperfusion. *Life Sciences* 252: 117679. 32325134 10.1016/j.lfs.2020.117679

[28] Bancroft, J.D. 1966. Observations on the effect on histochemical reactions of different processing methods. *Journal of Medical Laboratory Technology* 23 (2): 105–8 PMID: 5931043. 5931043

[29] Wang, J.S., et al. 2011. Celecoxib induces heme oxygenase-1 expression in macrophages and vascular smooth muscle cells via ROS-dependent signaling pathway. *Naunyn-Schmiedeberg’s Archives of Pharmacology* 383 (2): 159–168. 21174079 10.1007/s00210-010-0586-6

[30] Asghar, M., et al. 2021. Methotrexate Toxicity: A Simple Solution to a Complex Problem. *Cureus* 13 (4): e14364. 33972915 10.7759/cureus.14364 PMC8106237

[31] Yan, H., et al. 2021. Pharmacomicrobiology of Methotrexate in Rheumatoid Arthritis: Gut Microbiome as Predictor of Therapeutic Response. *Frontiers in Immunology* 12: 789334. 34975886 10.3389/fimmu.2021.789334 PMC8719371

[32] Jafaripour, L., et al. 2021. Effects of Rosmarinic Acid on Methotrexate-induced Nephrotoxicity and Hepatotoxicity in Wistar Rats. *Indian journal of nephrology* 31 (3): 218–224. 34376933 10.4103/ijn.IJN_14_20 PMC8330652

[33] Sherif, I.O., L.A. Al-Mutabagani, and O.M. Sarhan. 2020. Ginkgo biloba Extract Attenuates Methotrexate-Induced Testicular Injury in Rats: Cross-talk Between Oxidative Stress, Inflammation, Apoptosis, and miRNA-29a Expression. *Integrative Cancer Therapies* 19: 1534735420969814. 33118377 10.1177/1534735420969814 PMC7605049

[34] Bauerova, K., et al. 2015. Markers of inflammation and oxidative stress studied in adjuvant-induced arthritis in the rat on systemic and local level affected by pinosylvin and methotrexate and their combination. *Autoimmunity* 48 (1): 46–56. 25046647 10.3109/08916934.2014.939268

[35] Mahmoud, A.M., et al. 2017. Methotrexate hepatotoxicity is associated with oxidative stress, and down-regulation of PPARgamma and Nrf2: Protective effect of 18beta-Glycyrrhetinic acid. *Chemico-Biological Interactions* 270: 59–72. 28414158 10.1016/j.cbi.2017.04.009

[36] Calay, D., et al. 2010. Copper and myeloperoxidase-modified LDLs activate Nrf2 through different pathways of ROS production in macrophages. *Antioxidants & Redox Signaling* 13 (10): 1491–1502. 20446765 10.1089/ars.2009.2971

[37] Chang, M.Y., et al. 2010. AICAR induces cyclooxygenase-2 expression through AMP-activated protein kinase-transforming growth factor-beta-activated kinase 1–p38 mitogen-activated protein kinase signaling pathway. *Biochemical Pharmacology* 80 (8): 1210–1220. 20615388 10.1016/j.bcp.2010.06.049

[38] Jaiswal, A.K. 2004. Nrf2 signaling in coordinated activation of antioxidant gene expression. *Free Radical Biology & Medicine* 36 (10): 1199–1207. 15110384 10.1016/j.freeradbiomed.2004.02.074

[39] Ray, P.D., B.W. Huang, and Y. Tsuji. 2012. Reactive oxygen species (ROS) homeostasis and redox regulation in cellular signaling. *Cellular Signalling* 24 (5): 981–990. 22286106 10.1016/j.cellsig.2012.01.008 PMC3454471

[40] Ishikawa, K., and Y. Maruyama. 2001. Heme oxygenase as an intrinsic defense system in vascular wall: Implication against atherogenesis. *Journal of Atherosclerosis and Thrombosis* 8 (3): 63–70. 11866032 10.5551/jat1994.8.63

[41] Roghani, M., et al. 2020. Alleviation of Liver Dysfunction, Oxidative Stress and Inflammation Underlies the Protective Effect of Ferulic Acid in Methotrexate-Induced Hepatotoxicity. *Drug Des Devel Ther* 14: 1933–1941. 32546960 10.2147/DDDT.S237107 PMC7250701

[42] Matouk, A.I., et al. 2023. Dihydromyricetin Modulates Nrf2 and NF-kappaB Crosstalk to Alleviate Methotrexate-Induced Lung Toxicity. *Pharmaceuticals (Basel)* 16 (4): 481. 37111238 10.3390/ph16040481 PMC10145727

[43] Mansour, D.F., et al. 2021. Ginkgo biloba extract (EGb 761) mitigates methotrexate-induced testicular insult in rats: Targeting oxidative stress, energy deficit and spermatogenesis. *Biomedicine & Pharmacotherapy* 143: 112201. 34560547 10.1016/j.biopha.2021.112201

[44] Ozcicek, F., et al. 2020. Effects of anakinra on the small intestine mucositis induced by methotrexate in rats. *Experimental Animals* 69 (2): 144–152. 31787709 10.1538/expanim.19-0057 PMC7220717

[45] Arpag, H., et al. 2018. Protective Effects of Alpha-Lipoic Acid on Methotrexate-Induced Oxidative Lung Injury in Rats. *Journal of Investigative Surgery* 31 (2): 107–113. 28340320 10.1080/08941939.2017.1296513

[46] Hamdulay, S.S., et al. 2010. Celecoxib activates PI-3K/Akt and mitochondrial redox signaling to enhance heme oxygenase-1-mediated anti-inflammatory activity in vascular endothelium. *Free Radical Biology & Medicine* 48 (8): 1013–1023. 20083195 10.1016/j.freeradbiomed.2010.01.017

[47] Luo, C., et al. 2011. The role of COX-2 and Nrf2/ARE in anti-inflammation and antioxidative stress: Aging and anti-aging. *Medical Hypotheses* 77 (2): 174–178. 21530094 10.1016/j.mehy.2011.04.002

[48] Mammadov, R., et al. 2019. Effect of lutein on methotrexate-induced oxidative lung damage in rats: A biochemical and histopathological assessment. *Korean Journal of Internal Medicine* 34 (6): 1279–1286. 31495083 10.3904/kjim.2018.145 PMC6823580

[49] Kim, Y.J., M. Song, and J.C. Ryu. 2009. Inflammation in methotrexate-induced pulmonary toxicity occurs via the p38 MAPK pathway. *Toxicology* 256 (3): 183–190. 19100307 10.1016/j.tox.2008.11.016

[50] Drishya, S., S.S. Dhanisha, and C. Guruvayoorappan. 2022. Antioxidant-rich fraction of Amomum subulatum fruits mitigates experimental methotrexate-induced oxidative stress by regulating TNF-alpha, IL-1beta, and IL-6 proinflammatory cytokines. *Journal of Food Biochemistry* 46 (4): e13855. 34250612 10.1111/jfbc.13855

[51] Krause, M.M., et al. 2003. Nonsteroidal antiinflammatory drugs and a selective cyclooxygenase 2 inhibitor uncouple mitochondria in intact cells. *Arthritis and Rheumatism* 48 (5): 1438–1444. 12746918 10.1002/art.10969

[52] Yu, T., X. Lao, and H. Zheng. 2016. Influencing COX-2 Activity by COX Related Pathways in Inflammation and Cancer. *Mini Reviews in Medicinal Chemistry.* 16 (15): 1230–1243. 27145850 10.2174/1389557516666160505115743

[53] Sil, S., and T. Ghosh. 2016. Role of cox-2 mediated neuroinflammation on the neurodegeneration and cognitive impairments in colchicine induced rat model of Alzheimer’s Disease. *Journal of Neuroimmunology* 291: 115–124. 26857505 10.1016/j.jneuroim.2015.12.003

[54] Elmansy, R.A., et al. 2021. Rebamipide potentially mitigates methotrexate-induced nephrotoxicity via inhibition of oxidative stress and inflammation: A molecular and histochemical study. *Anatomical Record (Hoboken)* 304 (3): 647–661. 10.1002/ar.24482 32589351

[55] Younis, N.S., et al. 2021. Geraniol Averts Methotrexate-Induced Acute Kidney Injury via Keap1/Nrf2/HO-1 and MAPK/NF-kappaB Pathways. *Current Issues in Molecular Biology* 43 (3): 1741–1755. 34889889 10.3390/cimb43030123 PMC8929074

[56] Liu, Z., et al. 2024. Glutamine attenuates bisphenol A-induced intestinal inflammation by regulating gut microbiota and TLR4-p38/MAPK-NF-kappaB pathway in piglets. *Ecotoxicology and Environmental Safety* 270: 115836. 38154151 10.1016/j.ecoenv.2023.115836

[57] Doyle, T., et al. 2011. Intraplantar-injected ceramide in rats induces hyperalgesia through an NF-kappaB- and p38 kinase-dependent cyclooxygenase 2/prostaglandin E2 pathway. *The FASEB Journal* 25 (8): 2782–2791. 21551240 10.1096/fj.10-178095 PMC3136338

[58] Gong, P., B. Hu, and A.I. Cederbaum. 2004. Diallyl sulfide induces heme oxygenase-1 through MAPK pathway. *Archives of Biochemistry and Biophysics* 432 (2): 252–260. 15542064 10.1016/j.abb.2004.09.024

[59] Tanwar, L., V. Vaish, and S.N. Sanyal. 2009. Chemoprevention of 1,2-dimethylhydrazine-induced colon carcinogenesis by a non-steroidal anti-inflammatory drug, etoricoxib, in rats: Inhibition of nuclear factor kappaB. *Asian Pacific Journal of Cancer Prevention* 10 (6): 1141–1146. 20192600

[60] Fan, L.W., et al. 2013. Celecoxib attenuates systemic lipopolysaccharide-induced brain inflammation and white matter injury in the neonatal rats. *Neuroscience* 240: 27–38. 23485816 10.1016/j.neuroscience.2013.02.041 PMC3637873

