# Supplementary material for: Potential Effect of Etoricoxib in Reducing Inflammation in Methotrexate-Induced Pulmonary Injury in Rats: Role of Oxidative Stress and the TLR4/p38-MAPK/NF-κB Signaling Pathway
Source: Inflammation. 2024 Nov 27;48(4):2407–16. doi: 10.1007/s10753-024-02198-w (PMC12336082; doi:10.1007/s10753-024-02198-w)
Supplement: Supplementary file 1 — Supplementary file1 (PDF 129 KB) [file 10753_2024_2198_MOESM1_ESM.pdf]

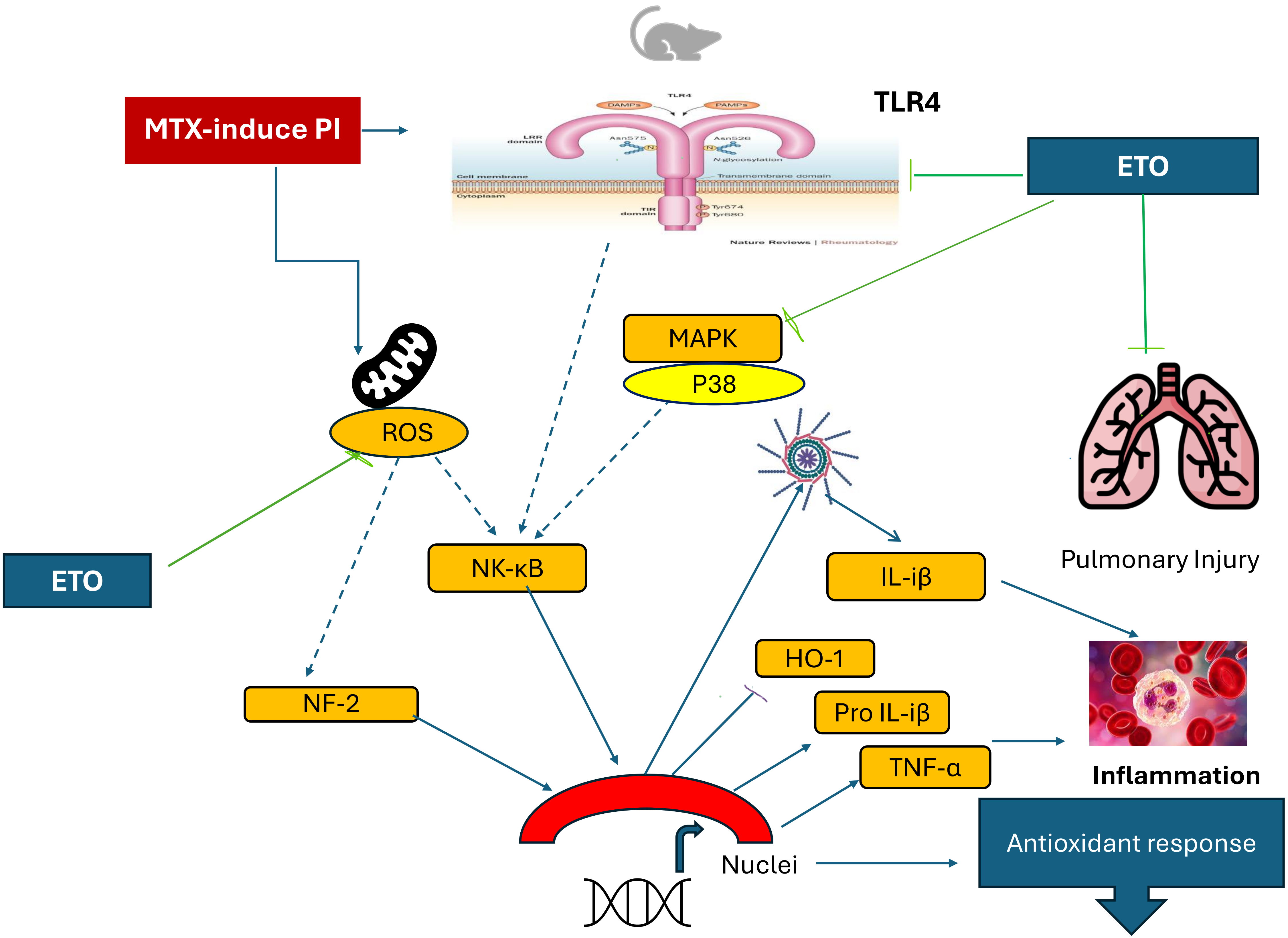
MTX-induce PI
TLR4
DAMPs
PAMPs
Cell membrane
Cytoplasm
Transmembrane domain
N-glycosylation
Nature Reviews | Rheumatology
ETO
MAPK
P38
ROS
ETO
NK-κB
IL-iβ
Pulmonary Injury
NF-2
HO-1
Pro IL-iβ
TNF-α
Inflammation
Antioxidant response
Nuclei
